# The network sustaining action myoclonus: a MEG-EMG study in patients with EPM1

**DOI:** 10.1186/s12883-016-0738-5

**Published:** 2016-11-07

**Authors:** Silvana Franceschetti, Laura Canafoglia, Fabio Rotondi, Elisa Visani, Alice Granvillano, Ferruccio Panzica

**Affiliations:** 1 Department of Neurophysiology, Epilepsy Centre, C. Besta Neurological Institute IRCCS Foundation, Via Celoria 11, 20133 Milan, Italy; 2 Department of Informatics, Bioengineering, Robotics and System Engineering (DIBRIS), University of Genova, Genova, Italy

**Keywords:** Action myoclonus, Cortico-muscular coherence, Cortical network, MEG

## Abstract

**Background:**

To explore the cortical network sustaining action myoclonus and to found markers of the resulting functional impairment, we evaluated the distribution of the cortico-muscular coherence (CMC) and the frequency of coherent cortical oscillations with magnetoencephalography (MEG). All patients had EPM1 (Unverricht-Lundborg) disease known to present with prominent and disabling movement-activated myoclonus.

**Methods:**

Using autoregressive models, we evaluated CMC on MEG sensors grouped in regions of interests (ROIs) above the main cortical areas. The movement was a repeated sustained isometric extension of the right hand and right foot. We compared the data obtained in 10 EPM1 patients with those obtained in 10 age-matched controls.

**Results:**

As expected, CMC in beta band was significantly higher in EPM1 patients compared to controls in the ROIs exploring the sensorimotor cortex, but, it was also significantly higher in adjacent ROIs ipsilateral and contralateral to the activated limb. Moreover, the beta-CMC peak occurred at frequencies significantly slower and more stable frequencies in EPM1 patients with respect to controls. The frequency of the beta-CMC peak inversely correlated with the severity of myoclonus.

**Conclusions:**

the high and spatially extended beta-CMC peaking in a restricted range of low-beta frequencies in EPM1 patients, suggest that action myoclonus may result not only from an enhanced local synchronization but also from a specific oscillatory activity involving an expanded neuronal pool. The significant relationship between beta-CMC peak frequency and the severity of the motor impairment can represent a useful neurophysiological marker for the patients’ evaluation and follow-up**.**

## Background

Neocortex is largely involved in the pathophysiology of movement disorders either directly, as a probable generator of particular types of myoclonus or rhythmic tremors or indirectly, by modulating the effect of subcortical generators. In pathological conditions, various studies investigated cortico-muscular coherence (CMC) between electroencephalography (EEG) or MEG and surface electromyography (EMG) signals with the aim of evaluating the relationship between cortical oscillations and EMG bursts. The consistent results of CMC analysis performed by Fast Fourier Transform or autoregressive (AR) methods in progressive myoclonic disorders [1–3] indicated that this approach is particularly effective in investigating the cortical origin of the jerks. Namely, this applies to the case of rhythmic myoclonus [2] or other subtle movement disorders such “cortical tremors” ([4, 5] for a review). Therefore, coherence analysis gained practical diagnostic value in rare or newly identified myoclonic syndromes, as well as in the examination of individual patients [6–8] Moreover, some observations also suggested a quantitative relationship between CMC values and the severity of the impairment resulting from this movement disorder [9].

Data obtained from patients with movement-activated jerks indicate that CMC between the sensorimotor region and the activated limb is mostly evident in beta-band, the same band in which CMC occurs during motor task in healthy subjects [10–12]. These observations suggest that “pathological” CMC in patients with jerky movement disorders may result from a purely quantitative distortion of the physiological CMC. However, the physiopathological mechanisms regulating the network responsible for enhanced CMC is still not clearly identified.

In this study we investigated the spreading of CMC using MEG signals recorded from patients with EPM1 (MIM #254800), a genetically determined neurological disorder characterized by prominent movement-activated myoclonus [13, 14]*,* therefore representing a disease “model” for this type of movement disorder. We performed this study with the aim of evaluating the extent and the topographical distribution of the brain cortico-muscular network involved in the generation of myoclonus and the frequency characteristics of the peak at which CMC occurs. Several studies have already established the presence of high coherence values between EEG or MEG activity and myoclonic EMG bursts [3, 15], but only a few of them compared the extent of the cortico-muscular network activated in healthy and pathological conditions during the same motor task [16]. MEG signals are suitable to perform this investigation due to the lower interference of superficial tissues and because MEG signals, in contrast to EEG, do not need any reference potentially susceptible to distort CMC amplitude and phase relationships. The final goal was the achievement of objective measures suitable to improve our understanding of the dysfunctional mechanism underlying myoclonus, to quantify the severity of motor impairment and, possibly, to find biomarkers able to monitor changes due to pharmacological and non-pharmacological treatments.

## Methods

We included ten patients with a diagnosis of EPM1 (three women) confirmed by the genetic finding of the homozygous expansion mutation of the CSTB gene or the compound heterozygous (expansion and point) mutation. The mean disease duration in EPM1 patients was 29.2 ± 4.7 years. The predominant symptom in all of the patients was myoclonus, whereas mental decline was minimal or absent; no subjects had neurological comorbidities. Myoclonus severity was scored according to a simplified functional scale with five degrees [17] (Table 1). All patients received an anti-myoclonic and antiepileptic treatment always including valproate. Only three of them received low doses of benzodiazepines (Table 1). This pharmacological therapy was able to control seizures in all of the patients. We compared the results of the MEG–EMG analyses of the EPM1 patients with those obtained in 10 healthy (six women) volunteers who underwent the same type of neurophysiological examination. The mean age at the time of the MEG recordings was similar in EPM1 and controls (43.3 ± 15.4 years and 40.3 ± 15.9 years).

**Table 1:** Main parameters of the disease and disability resulting from myoclonus; pharmacological treatment

Patient N, gender	Age (years)	Disease Onset (years)	Disease Duration (years)	Score	Treatment (daily dose in mg)
#1, F	28	11	17	2	VPA 1200, LEV 1000, TPM 200
#2, M	27	16	11	1	VPA 1750, LEV 3000
#3, M	43	14	29	1	VPA 1500, ZNS 200
#4, M	52	17	35	1	VPA 2400, PB 50
#5, F	23	11	12	2	VPA 1150, LEV 1750
#6, M	54	15	39	2	VPA 2500, ZNS 200
#7, M	50	16	34	4	VPA 1800, CZP 1, TPM 300, ZNS 500
#8, M	60	16	44	3	VPA 1200, PB 150, CZP 1
#9, F	70	14	56	5	VPA 1300, LEV 2000, TPM 100, CZP 4
#10, F	26	11	15	3	VPA 1750, ZNS 300

Legend: Score: assessed according to a simplified functional scale [17]: treatment: *VPA* valproate, *TPM* topiramate, *LEV* levetiracetam, *ZNS* zonisamide, *PB* phenobarbital, *CZP* clonazepam

We recorded MEG signals in a magnetically shielded room (VACUUMSCHMELZE GmbH & Co KG, Hanau Germany) with a 306-channel whole head MEG system (Neuromag Triux, Elekta Oy, Sweden). Signals were filtered in the band 0.1-330 Hz and sampled at 1 kHz. Bipolar electro-oculographic and electrocardiographic signals were acquired in order to monitor and remove ocular and cardiac artefacts. Subjects laid in supine position with eyes closed.

The subject’s head position inside the MEG helmet was continuously monitored by five head position identification (HPI) coils located on the scalp. The locations of these coils, together with three anatomical landmarks (nasion, right and left preauriculars), and additional scalp points were digitized before the recording by means of a 3D digitizer (FASTRAK, Polhemus, Colchester, VT).

Surface EMG signals were simultaneously recorded from pairs of electrodes placed bilaterally 2–3 cm apart over the belly of the right and left flexor and extensor wrist muscles and of right tibialis anterior and gastrocnemius.

MEG and polygraphic signals were recorded at rest, and during periods of maintained right wrist or foot extension (five sequences of 1 min each).

### Data processing

MEG signals were pre-processed off-line with the temporally extended signal space separation method (tSSS, [18]) implemented in the Maxfilter 2.2 (Elekta Neuromag Oy, Helsinki, Finland) to suppress external interferences and correct for head movements, and next filtered in the 1.6-100 Hz band with a zero phase digital filter.

The MEG system used for this study is endowed with 204 planar gradiometers (102 with derivative along longitude (y-axis) and 102 along latitude (x-axis), and 102 magnetometers. In this study, we used the planar gradiometers, which are mainly sensitive to sources close to the sensor array, and relatively insensitive to homogenous fields; moreover, the topographic maps obtained from gradiometers show maxima just over the sources, whereas they show patterns with one positive and one negative maxima symmetrically arranged perpendicularly to the source axis when obtained from magnetometers. As far the type and combinations of gradiometers, different approaches were used in previous connectivity studies. Some authors considered the whole set [19], whereas others chose either to select the orientation showing the highest connectivity values [20], or a combined value of each gradiometer couples [21, 22]. We chose to report results obtained from the set of 102 gradiometers with derivative along the y-axis (y-gradiometers), since they showed in all of the subjects a clearer and more stable CMC response during the voluntary movements, in particular over the sensorimotor cortex, with respect to gradiometers with derivative along the x-axis.

CMC was estimated by means of a blockwise bivariate AR model. The AR model order was determined using the multichannel version of the Akaike criterion as a guideline and the goodness of the identification was verified by means of ‘portmanteau’ chi-square and Anderson’s tests [23, 24]. Coherence was defined as:

$$ Co{h}_{xy}(f)=\frac{{\left|{C}_{xy(f)}\right|}^2}{S_{xx}(f){S}_{yy}(f)} $$

where S_xx_ (f) and S_yy_ (f) are the power spectral densities of the MEG and EMG channels, and S_xy_ (f) is the cross-spectral density. The critical value for the null hypothesis of zero-coherence at a significance level of 0.01 was computed according to [25], taking into account that the degree of freedom of an AR model is given by N/p [26], where N is the number of samples, p the model order, and that the asymptotic variance of the AR spectral estimate is similar to that of the smoothed periodogram with the same number of degrees of freedom [27, 28].

About three minute of the MEG and EMG signals free from artefacts was selected for the analysis, normalised by subtracting the mean value and dividing the result by the standard deviation, and then divided into non-overlapping 1-s epochs. The epochs were considered multiple realisations of the same process and the autoregressive coefficients were estimated by means of the Levinson-Robinson-Wiggins algorithm [29].

In order to evaluate CMC patterns in different areas, the sensors were grouped into 12 regions of interest (ROIs): right (RF), and left (LF) frontal, right (RLP) and left (LLP) lateral parietal, right (RO) and left (LO) occipital, frontal vertex (FV), occipital vertex (OV), left and right parietal vertex (VLP, VRP), left and right temporal (LT, RT), according to the layout of sensor elements, and the CMC within each ROI was averaged.

All of the analyses were made using custom-written routines in Matlab (Version 8.3, R2015b; Mathworks Inc., Natick, MA, USA), which also contained modified functions from the Biosig toolbox [30].

### Statistical analysis

Fisher’s Z transformation was applied to CMC values in order to normalize their distribution. The data were statistically analyzed using the SPSS software (version 17, SPSS Inc. Chicago, IL, USA). We used repeated measures analysis of variance (RM-ANOVA) at a significance level of 5 % to assess the effects of the between group (EPM1 patients and controls) and the within-group (mean beta-CMC coherence in ROIs) factors. The sphericity assumption was evaluated using Mauchley’s test, and the Greenhouse-Geisser correction was applied when appropriate. When RM-ANOVA showed significant main effects or interactions, post hoc analysis (ANOVA) was used. We applied non-parametric U-Mann or Wilcoxon tests to compare the ordinal values (e.g. number of gradiometers), and the Pearson’s correlation analysis to evaluate the relationship between the CMC peak magnitude or frequencies and clinical parameters.

## Results

During the execution of both upper and lower limb motor tasks, all subjects showed significant beta-CMC on more than one sensor exploring parietal and/or frontal areas.

### CMC magnitude and distribution

Comparing EPM1 with controls, RM-ANOVA showed significant “between subjects” differences for the mean beta-CMC magnitude during both upper (F (1,18) = 12.24, *p* = 0.003) and lower limb (F (1,18) = 4.8, *p* = 0.034) motor task.

**Fig. 1:**
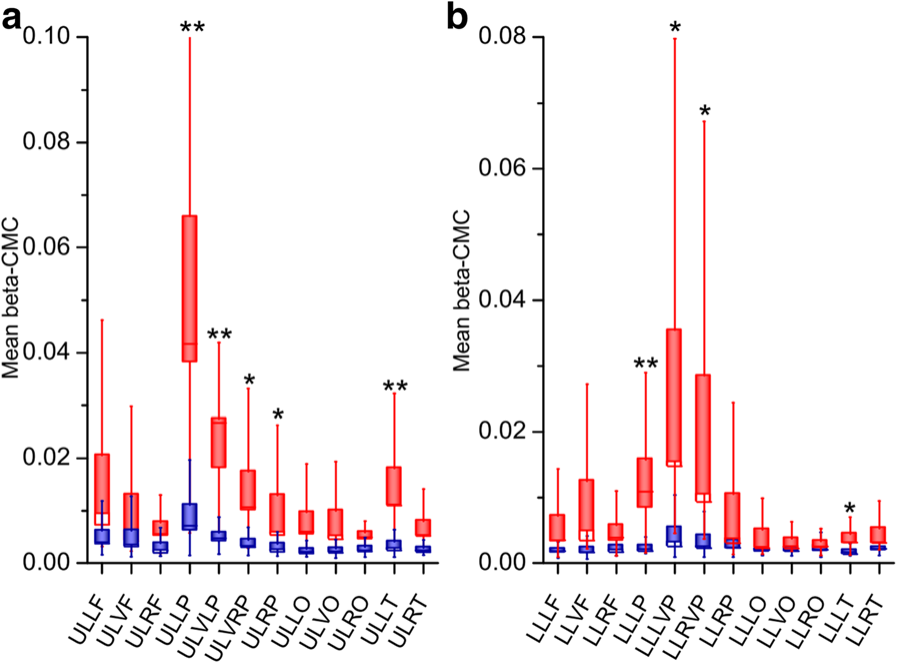
Beta-CMC magnitude during the motor task. Each box represents the mean values and the SEM measured in EPM1 patients (in red) and controls (in blue) during upper (**a**) and lower limb (**b**) motor tasks; whiskers represent the 10–90 % of the maximal values; * = *p* < 0.05; ** = *p* < 0.01; UL: upper limb, LL: lower limb, LF: left frontal, VF: vertex frontal, RF: right frontal, LP: left parietal, VLP: vertex left parietal, VRP: vertex right parietal, RP: right parietal, LO: left occipital, VO: vertex occipital, RO: right occipital, LT: left temporal, RT: right temporal)

For the right upper limb motor task, post-hoc tests performed comparing different ROIs, showed significant differences between EPM1 patients and controls on those ROIs including the brain areas physiologically activated during the motor task (e.g. lateral parietal ROIs) (Fig. 1a), but also the bilateral parietal paramedian (vertex) and the left temporal ROI. During the right lower limb motor task, significantly higher beta-CMC was observed in EPM1 patients in sensors located on left lateral parietal, bilateral vertex ROIs and left temporal ROI (Fig. 1b).

**Fig. 2:**
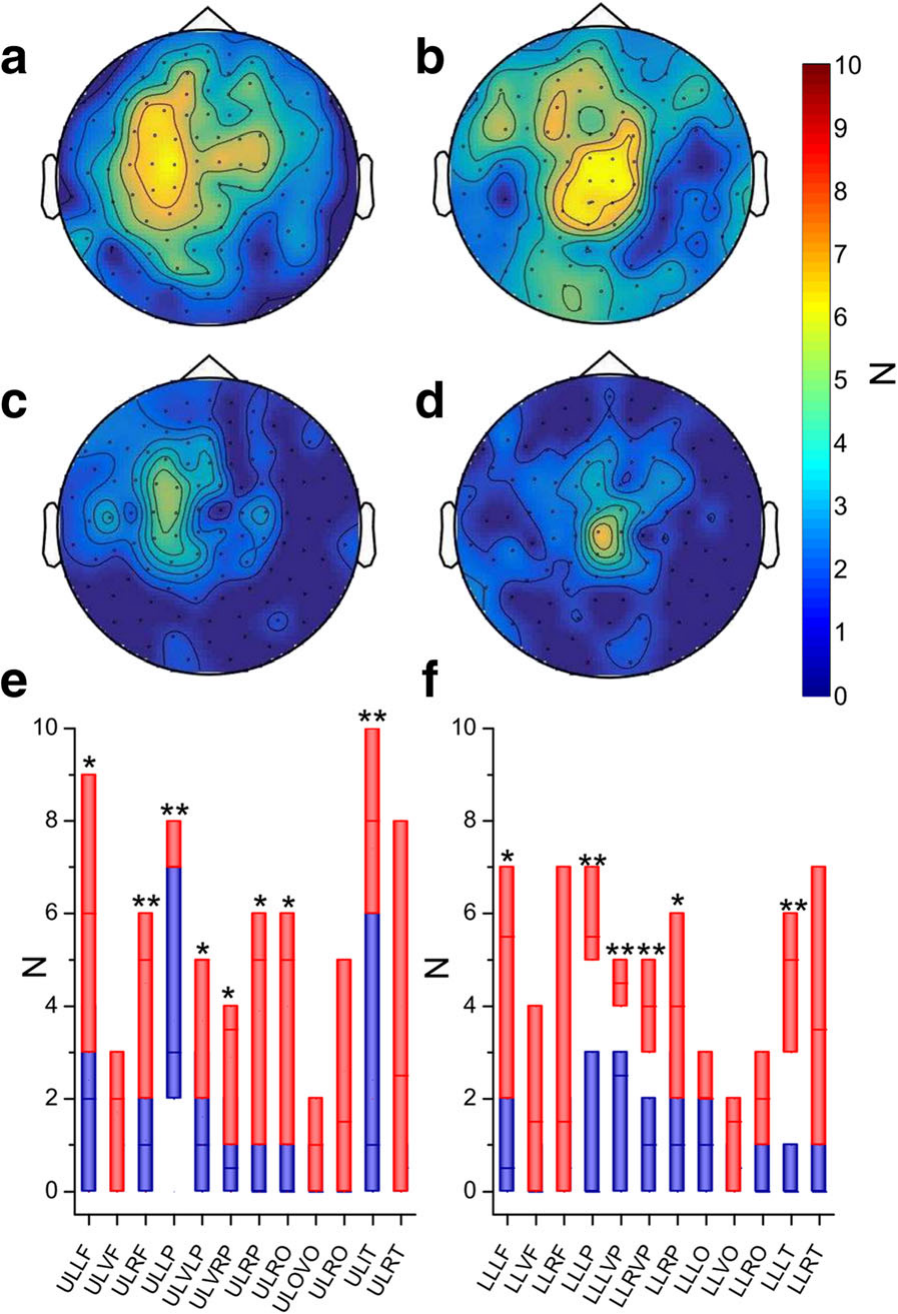
Y-gradiometers with supra-threshold beta-CMC during the motor task. Topographical representation of MEG sensors number showing significant beta-CMC in EPM1 patients and controls during upper limb (**a** and **c**, UL) and lower limb (**b** and **d**, LL) motor tasks. Plots **e** and **f** display the number of sensors in ROIs between EPM1 patients (in red) and controls (*in blue*) (each box represents the 25° and 75° percentile). (* = *p* < 0.05; ** = *p* < 0.01). Abbreviations as in Fig. 1

Overall, the number of y-gradiometers showing supra-threshold beta-CMC was significantly higher in EPM1 patients than in the controls for the upper limb (median 51.0, Q1 26.75 vs. median 10.0, Q1 4.75; *p* < 0.001; Fig. 2a and c) and for the lower limb (median 42.0, Q1 31.00 vs. median 11.0, Q1 6.0; *p* = 0.001; Fig. 2b and d).

Post-hoc tests indicated that the number of gradiometers showing supra-threshold beta-CMC was higher in EPM1 patients also in the ROIs not showing significantly higher mean beta-CMC values, including bilateral frontal ROIs, and left occipital ROIs both in case of upper limb and lower limb motor task (Fig. 2e and f).

### Frequency of CMC peaks

**Fig. 3:**
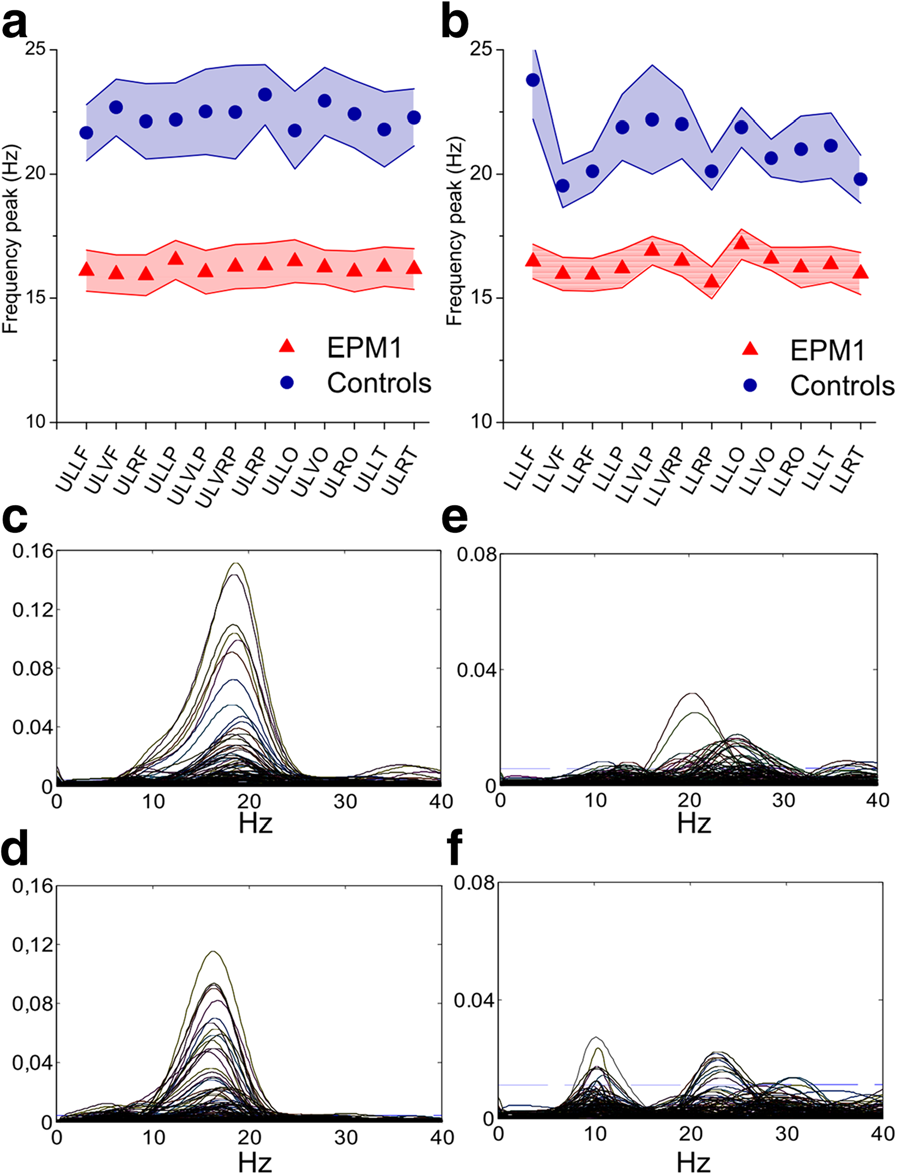
Frequency of beta-CMC peaks during the motor task. Beta-CMC peak frequency in EPM1 patients and controls during the upper (**a**, UL) and lower (**b**, LL) limb motor tasks in the different macro areas. The shaded areas represent the standard error. Panels (**c**-**f**) display representative examples of CMC spectra on all sensors measured in two EPM1 patients (#1 and #3, **c** and **d**) and in two controls (**e** and **f**). Abbreviation as in Fig. 1

The mean frequency of beta-CMC peaks was significantly lower in EPM1 patients than in controls during both upper (16.19 ± 0.71 vs. 22.28 ± 1.24; F (1,18) = 17.23 *p* < 0.001) and lower limb (16.34 ± 0.62 Hz vs. 21.11 ± 0.9.0 Hz; F (1,18) = 20.34, *p* < 0.001) motor task. As shown in Fig. 3a and b, EPM1 patients showed quite uniform CMC peak frequencies during both upper and lower limb tasks, while controls showed a more scattered frequency distribution. Moreover, intra-subject variability was lower in EPM1 patients than in controls, as testified by the smaller standard error (shaded areas in Fig. 3a and b). Therefore the peak frequency was rather consistent in individual EPM1 patients but variable in individual controls, as exemplified in the panels c-f of Fig. 3, showing the mean coherence spectra obtained from all y-gradiometers during the upper limb motor task in two representative EPM1 patients (c and d) and in two controls (e and f).

Both EPM1 patients and controls also showed a CMC peak in the theta-alpha band (from 6 to 13 Hz), whose mean frequency was slower in EPM1 patients (see examples in Fig. 3e and f (upper limb 6.7 ± 0.23 Hz vs. 9.26 ± 0.33 Hz, *p* < 0.001; lower limb 6.70 ± 0.23 Hz vs. 9.36 ± 0.33 Hz; *p* < 0.001). Considering the ROIs primarily involved in CMC (LF, LLP, VLP, LT) we found that, both in case of upper and lower limb motor tasks, the mean theta-alpha-CMC value was significantly lower than those of beta-CMC in EPM1 patients, while this not occurred in controls, (upper limb: 0.004 ± 0.001 vs. 0.026 ± 0.006 in EPM1 patients, *p* = 0.008 and 0.003 ± 0.001 vs. 0.005 ± 0.001 in controls) (lower limb: 0.004 ± 0.001 vs. 0.013 ± 0.004, *P* = 0.009 in EPM1 patients and, 0.003 ± 0.001 vs. 0.003 ± 0.001 in controls).

### Relationship between MEG-EMG coherence and clinical parameters

In controls, we did not find any significant relationship between age at the time of MEG recording and beta-CMC magnitude, number of y-gradiometers showing supra-threshold beta-CMC or frequency of beta-CMC peaks. In EPM1 patients, the magnitude of beta-CMC evaluated on the left parietal ROI during the right upper limb task slightly decreased with age (which paralleled the duration of the disease) (rho = −0.790, *p* = 0.007).

**Fig. 4:**
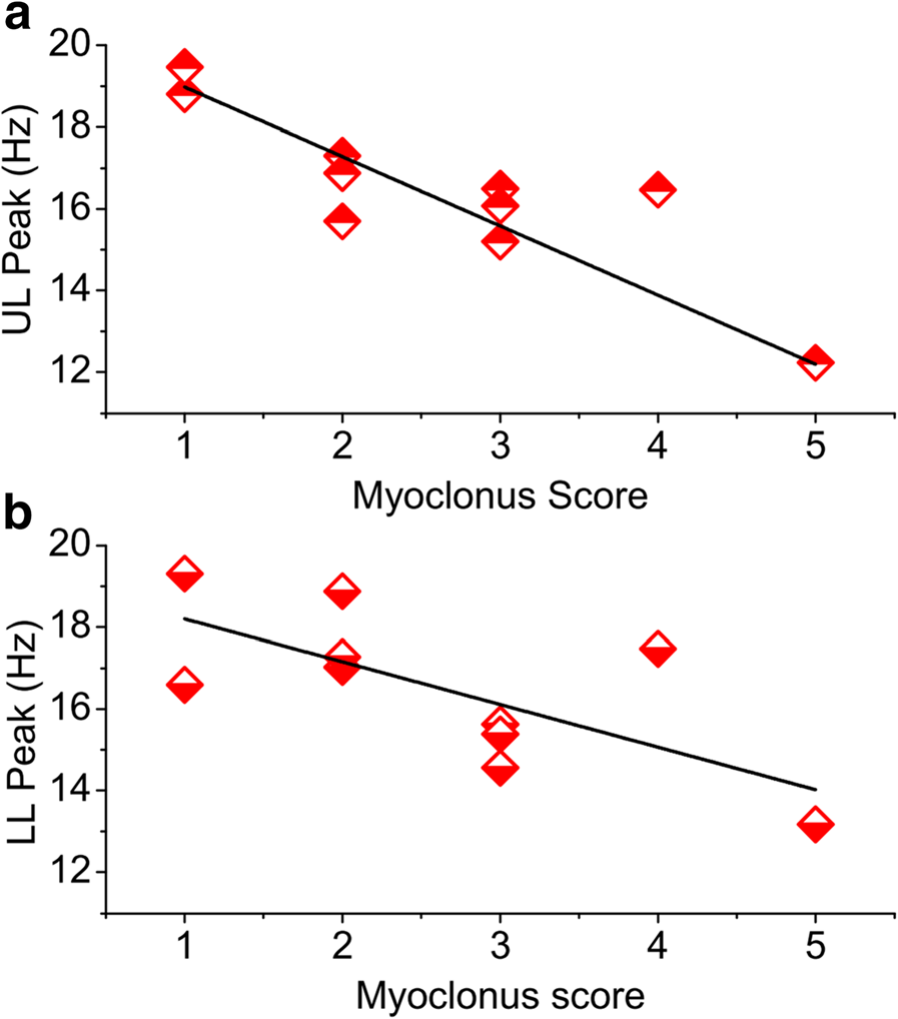
Relationship between beta CMC peaks and severity of myoclonus. Plot of the coherent beta-CMC peak mean frequency during upper limb (UL) (**a**) and lower limb (LL) (**b**) motor task vs. functional score, describing the severity of myoclonus in EPM1 patients. Lines represent the linear fitting of the mean values measured in each patient

In EPM1 patients, the severity of myoclonus (Table 1) was not explicitly related to age or disease duration (rho = 0.503, *p* = 0.139). By evaluating the relationship between myoclonus severity and beta-CMC peaks, we found that only the beta-CMC peak frequency, but not its magnitude, significantly correlated with the myoclonus functional score. During the upper limb motor task, this was true either considering the overall mean frequency values (rho = 0.843; *p* = 0.002) (Fig. 4), or frontal, parietal and left temporal ROIs, independently. During the lower limb motor task we found a similar relationship between the overall mean values of beta-CMC frequency and severity of myoclonus (rho = 0.698; *p* = 0.025) but not for individual ROIs.

## Discussion

Our results, indicating significantly higher beta-CMC values in EPM patients compared to controls agree with previous findings obtained in patients with progressive myoclonus epilepsies both on EEG and MEG data [1–3, 15], but they also reveal the presence of significant beta-CMC involving other regions besides parietal and frontal areas contralateral to the activated limb, and point out a significant shift of the beta CMC peak towards low beta frequencies in EPM1 patients.

Our evidences agree with those obtained by [15] who found that the amplitude of the dominant coherent peaks were 2–4 fold compared with the healthy controls and reported an additional ipsilateral coherent activity in the majority of patients. Furthermore, our findings indicate that the cortical area involved in the coherent network is more extended with respect to sensorimotor cortex contralateral to activated limb and consistently includes ipsilateral sensorimotor cortex and sensors located in adjacent cortices. We consider that an important and new finding is the coupling between peculiar low-beta frequencies of the dominant coherent peak with abnormally high CMC, a peculiar characteristic of myoclonus-related oscillations and correlate with motor disability.

The observation of a significant CMC distribution on wider cortical areas is also in line with our previous study performed on EEG using the generalized partial directed coherence [16], where we found that patients with EPM1 consistently have a robust outflow towards the activated muscles compared to controls, involving, besides the contralateral hemisphere, the one ipsilateral to the activated limb. Likewise, we previously found in EPM1 patients a widening of the cortical areas participating in the generation of pre-movement alpha-desynchronization [31].

In the present study, the use of high density MEG sensors and the absence of any signal reference issue, differently from EEG signals, certainly contributed in revealing the great extent of the synchronization pattern involving contralateral premotor and temporal areas, together with sensorimotor areas ipsilateral to the activated limb.

The involvement of pre-motor and ipsilateral motor cortices into the pathophysiology of segmental action myoclonus found in EPM1 patients while performing a motor task may derive from a “compensatory” phenomenon, as previously assumed for patients with other type of motor dysfunctions [32]. However, it can also reveal that the mechanism sustaining action myoclonus originates from intrinsic behaviour and extent of the oscillating neuronal pool. Indeed, an important finding concerns the frequency of the myoclonus-related CMC peak in beta-band, which was consistently and significantly slower in EPM1 patients compared to that estimated during muscle contraction in healthy subjects; moreover, in patients the CMC peaks occurred in a narrow frequency range, whereas in controls it occurred at more scattered frequencies.

These findings suggest that myoclonus does not result from merely enhanced CMC in beta-band, but from oscillations occurring in a restricted frequency range attributable to a specific rearrangement of the involved neuronal pool. We found similar slowing in the beta event-related synchronization/desynchronization (ERD-ERS) during simple motor tasks [31, 33].

Silén et al. 2000 [34] reported a slower CMC peak frequency in beta band in patients with EPM1 studied with MEG and ascribed this slowing to degenerative changes in the brain or to drug treatment. In healthy subjects, MEG studies showed that the frequency of beta oscillations tends to decrease (whereas power increase) with the administration of benzodiazepine [35] or with aging [36]. In our study, we did not observe an age-related change in beta-CMC magnitude or peak frequency in healthy controls. Actually, our groups included younger subjects, on average, than those studied by [36]; thus, it is possible that we did not capture these variations. As well, even if our patients took antiepileptic drugs, only three of them took benzodiazepines. Thus, we have no reasons to consider that the pharmacological treatment played a relevant role in slowing the beta-CMC peak frequency. The coherent beta-CMC peak, in fact, was not only significantly slower in patients than in controls but also showed a limited variability among different patients. Moreover, we found a significant association between the beta-CMC peak frequency and the severity of myoclonus.

The lower beta-CMC peak frequency found in EPM1 patients, and its consistency along many sensors and epochs, indicates that this measure can be a useful biomarker to evaluate the degree of the dysfunctional cortical network in PME. Indeed, the beta-CMC peak frequency found in our EPM1 patients was lower (or in a lowest beta range) compared to the values observed not only in our control group, but with values found in healthy subjects reported in other studies. In fact, healthy peak frequencies measured during isometric muscle contraction commonly range from 15 to 30 Hz [2, 10, 11, 37, 38], and only a few studies detected slower frequencies (6–15 Hz) in some individuals [37].

In addition to beta-CMC, we also found coherent peaks in theta-alpha band, in agreement with findings obtained from healthy subjects [12, 39]. In our controls, theta-alpha-CMC was significantly higher than in EPM1 patients. We can just hypothesize that in controls this theta-alpha-CMC represents a residual mu component persisting in spite of the motor activity.

Most data obtained in patients and in the animal model of EPM1 [40] support a loss of GABA inhibition. In fact, both transcranial magnetic stimulation protocols [41, 42] on patients, and experiments performed on the EPM1 mouse model indicated reduced intracortical GABA-mediated inhibition and loss of GABA-interneurons [43, 44]. However, other data, obtained in a large group of EPM1 patients, conversely support an increased GABA inhibition, possibly deriving from a compensatory mechanism to counteract hyperexcitability [45].

Interneurons are anyway implied not only in local feedback inhibition but, directly, in the generation and modulation of cortical oscillations [46], and the effect of GABA agonists on oscillatory networks appears to be not simply predictable. For instance, the effect of GABA agonists seems to be both dose dependent [47] and affected by receptor desensitization, thus making difficult to infer the final effect of GABA in complex oscillatory networks. Moreover, recent data proposed that beta activity reactive to movement could have a different origin, partially independent from GABA inhibition [48]. Therefore, our hypothesis of a peculiar oscillatory state occurring in neocortical areas as responsible for the generation of action myoclonus is not conflicting with previously described or hypothesized GABA-dependent mechanisms. Moreover, the present observation could be in line with the hypothesis declaring that hypersynchronous oscillations in beta-band limit the ability of neurons to code information in time and space, and thus sustains myoclonic bursts [49]*.*

The observation of a significant relationship between the frequency of the beta-CMC peak and the severity of myoclonus on both upper and lower limb (impairing motor ability and autonomous walk), independently from the disease duration, may also suggest that the coupling between significantly high CMC, low-beta peak frequency and functional movement impairment can represent a useful marker for the neurophysiological evaluation and follow-up of EPM1 patients during pharmacological or rehabilitative treatments. This relationship was more obvious for the upper limb than for the lower limb. It is possible that this derives from the higher complexity of the cortical control of the upper limb, more influenced by distorted cortical oscillations in comparison with the more simple and automatic scheme of the lower limb motility.

A limitation of our study derives from the fact that we evaluated the extent of the area showing MEG-EMG coherent beta oscillations at sensor level. Magnetic field spread can cause smearing of the effect of the neural generators at the surface and induce spurious correlations between MEG sensors. An analysis at source-space level should thus enhance the spatial resolution of our study. However, in our study we compared the coherence patterns obtained from EPM1 patients versus controls and evaluated their differences from a statistical point of view. Therefore, we expect that if our CMC results resulted inflated or smeared by magnetic field spread, this was also true for the subject groups controls in a comparable way, thus limiting this type of bias. To better understanding the role of cortical regions outside the contralateral sensorimotor cortex and their involvement in generating myoclonus, further studies evaluating both CMC and cortico-cortical effective connectivity and network topology may add useful information.

## Conclusions

Our observation shows that the strongly enhanced beta-CMC found in EPM1 patients during cortical myoclonus does not represent a merely quantitative phenomenon due to increased neuronal synchronization but resides on a disease-dependent change of the neuronal network. Actually, in EPM1 patients enhanced beta-CMC occured in an enlarged cortical area, but in a restricted range of low-beta frequencies while in controls beta-CMC involved broader and less stable frequencies. The significant relationship between beta-CMC peak frequency and the severity of the motor impairment discloses the degree of pathological network reorganization and may represent a useful neurophysiological marker for the patients’ evaluation and follow-up.

## Abbreviations

CMC: Cortico-muscular coherence
EEG: Electroencephalography
EMG: Electromyography
MEG: Magnetoencephalography
ROI: Region of interest
